# Human infection challenge studies in endemic settings and/or low-income and middle-income countries: key points of ethical consensus and controversy

**DOI:** 10.1136/medethics-2019-106001

**Published:** 2020-05-07

**Authors:** Euzebiusz Jamrozik, Michael J Selgelid

**Affiliations:** 1 Monash Bioethics Centre, Monash University, Melbourne, Victoria, Australia; 2 Department of General Medicine, Royal Melbourne Hospital, Melbourne, Victoria, Australia

**Keywords:** research ethics, research on special populations, Communicable disease

## Abstract

Human infection challenge studies (HCS) involve intentionally infecting research participants with pathogens (or other micro-organisms). There have been recent calls for more HCS to be conducted in low-income and middle-income countries (LMICs), where many relevant diseases are endemic. HCS in general, and HCS in LMICs in particular, raise numerous ethical issues. This paper summarises the findings of a project that explored ethical and regulatory issues related to LMIC HCS via (i) a review of relevant literature and (ii) 45 qualitative interviews with scientists and ethicists. Among other areas of consensus, we found that there was widespread agreement that LMIC HCS can be ethically acceptable, provided that they have a sound scientific rationale, are accepted by local communities and meet usual research ethics requirements. Unresolved issues include those related to (i) acceptable approaches to trade-offs between the scientific aim to produce generalisable results and the protection of participants, (iii) the sharing of benefits with LMIC populations, (iii) the acceptable limits to risks and burdens for participants, (iv) the potential for third-party risk and whether the degree of acceptable third-party risk is different in endemic settings, (v) the conditions under which (if any) it would be appropriate to recruit children for disease-causing HCS, (v) appropriate levels of payment to participants and (vi) appropriate governance of (LMIC) HCS. This paper provides preliminary analyses of these ethical considerations in order to (i) inform scientists and policymakers involved in the planning, conduct and/or governance of LMIC HCS and (ii) highlight areas warranting future research. Insofar as this article focuses on HCS in (endemic) settings where diseases are present and/or widespread, much of the analysis provided is relevant to HCS (in HICs or LMICs) involving pandemic diseases including COVID19.

## Introduction

Human infection challenge studies (HCS) involve intentionally infecting research participants with pathogens (or other micro-organisms) with the primary aim(s) of (i) testing (novel) vaccines and/or therapeutics, (ii) generating knowledge regarding the natural history of infectious diseases (and/or asymptomatic infection) and/or (iii) developing ‘models of infection’—that is, reliable methods (to be used in studies with aims (i) or (ii)) of infecting participants with particular micro-organisms). Modern HCS are sometimes referred to as ‘controlled human infection studies’ because they involve *controlling* the selection and/or production of the micro-organism strain and the timing, route and/or dose of infection; infection in a *controlled* environment; infection with micro-organisms causing no disease or disease that is self-limiting and/or can be (and is) *controlled* with early diagnosis and/or effective cures/treatments; and/or *controlling* who is being infected (and/or subjected to other experimental interventions).

HCS can accelerate the development of new vaccines (and therapeutics) because they can be substantially smaller, shorter and less expensive than other kinds of studies. Among other advantages, far fewer people need to be given experimental vaccines (that might not turn out to be safe or effective) in HCS than in field trials. The latter are much larger, and commonly involve thousands of participants; whereas most HCS involve less than 100 participants. HCS can also provide unique insights in to host-pathogen interactions.^1 2^

Although several prominent historical cases of unethical research involved the intentional infection of research participants,^3–5^ HCS have in recent decades been conducted with research ethics oversight and careful research practices, collectively enrolling tens of thousands of consenting healthy volunteers.^1^ Although the vast majority of the burden of infectious diseases occurs in low-income and middle-income countries (LMICs), modern HCS have until recently been conducted almost exclusively in high-income countries (HICs). In fact, around 99% of the >40 000 human volunteers who have participated in HCS worldwide since World War II have been in HICs.^6–8^ There have been increasing calls for HCS in LMICs, and such studies are being increasingly conducted, in part because findings from research on HIC volunteers are often less relevant to at-risk populations in LMICs (eg, due to population differences regarding naturally acquired immunity, co-infections, genetics, microbiome, nutrition, etc).^1^ ^9^

Despite the potential scientific advantages of (endemic and/or LMIC) HCS, such studies are nonetheless ethically sensitive. HCS raise ethical issues involving controversial aspects of research ethics more generally (eg, regarding benefit sharing, limits to risk, the right to withdraw, informed consent, payment of participants and research with children or other ‘vulnerable’ participants), but they also raise ethical issues that are more specific to HCS in particular (eg, third-party risks related to challenge infections and the potential need for special ethical and/or regulatory governance mechanisms).

This paper summarises the findings of a project that examined the ethical and regulatory issues related to HCS in endemic and/or LMIC settings via (i) a review of relevant literature and (ii) qualitative interviews involving 45 scientists and ethicists with relevant expertise (table 1). Details of the search strategy and qualitative interview methodology can be found in the online supplementary appendix, which also contains qualitative data (ie, quotations from expert interviews) as a resource that is intended to enrich the preliminary analyses summarised in the main body of this article. Below, we highlight (i) areas of consensus among the experts we interviewed and/or in the research ethics literature regarding issues that are highly salient to challenge studies, as well as (ii) contentious and/or unresolved issues warranting further analysis and/or particularly careful attention during the design and conduct of such studies.

10.1136/medethics-2019-106001.supp1 Supplementary data

Insofar as this article focuses on HCS in (endemic) settings where diseases are present and/or widespread, much of the analysis provided is relevant to HCS (in HICs or LMICs) involving pandemic diseases including coronavirus disease 2019 (COVID19).

## Ethical issues

### Intentional infection

In the limited ethics literature on HCS to date, there appears to be a consensus that intentional infection of research participants (and the risks associated with it, once appropriately minimised) can be permissible so long as certain ethical conditions are met.^8 10–15^ ^2^ Experts interviewed as part of our qualitative research were likewise unanimous in considering intentional infection of research participants to be an ethically acceptable research practice, at least under certain conditions. Nevertheless, many stakeholders suggested that HCS warrant particularly careful ethical and scientific evaluation because (i) well-designed HCS offer potentially significant scientific benefits and social value, (ii) healthy individuals who volunteer for HCS often bear significant risks and/or other burdens related to study participation and (iii) public acceptance of HCS may be contingent on such studies being conducted with a high degree of safety.

## Benefits

### Potential ethical imperative to conduct LMIC HCS

Given that HCS are often expected to lead to significant public health benefits more efficiently (eg, in terms of time, costs and number of research participants) than alternative research designs,^2 16^ some commentators have argued that, beyond such studies being merely ethically acceptable, there is an ethical imperative to conduct HCS if (or when) no other (less burdensome) feasible research design could obtain similarly valuable results and if (or when) *not* performing HCS could lead to greater net harms including (i) longer delays to the development and implementation of beneficial new interventions for infectious diseases and/or (ii) the exposure of more participants to potentially greater risks in alternative study designs (eg, field trials). Such considerations might especially favour HCS for diseases that cause persistently high annual burdens of disease (eg, malaria) and/or emerging diseases (eg, COVID19). 11 15

If there is an ethical imperative to conduct HCS (in general) that is grounded (in part) in the need to relieve significant burdens of infectious disease, then there is arguably an even stronger ethical imperative to conduct HCS in endemic settings and/or LMICs in particular because, *inter alia*, (i) HCS in endemic LMICs may be more scientifically valid and/or efficient in terms of producing results that are relevant to disease control in at-risk populations, (ii) in at least some cases participants in endemic LMIC HCS might benefit directly from research participation (ie, they end up with immunity to a disease to which they are at risk via a less harmful bout of illness than may have otherwise occured or via inoculation with an experimental vaccine that turns out to be effective) 3 15 ^3^, (iii) endemic-region HCS are more likely to recruit individuals drawn from populations that stand to benefit from (any interventions developed as a result of) the research (ie, the burdens and the benefits of the research occur in the same or similar populations), (iv) HCS may be less risky to some participants in endemic regions and (v) building local capacity for (infectious disease) research in LMICs may help to increase the degree to which research addresses neglected diseases (which are predominantly endemic in LMICs).^18^

There are thus strong ethical reasons that support conducting (more) endemic and/or LMIC HCS. However, even if there is an especially strong case for conducting such studies, certain (other) ethical issues related to their design and conduct warrant particularly careful attention. This is because (i) HCS may sometimes involve, or at least be perceived to involve, particularly high levels of risks (for participants and third parties) and other burdens for participants (and such studies must therefore be carefully designed and conducted to ensure that expected benefits outweigh risks and burdens), (ii) local and/or international community acceptance of HCS being conducted in endemic LMICs may be contingent on such studies being designed and conducted to especially high ethical (and scientific) standards and (iii) certain ethical considerations, although familiar in research ethics discourse, may have particular (underexplored) implications in the context of endemic LMIC HCS. The evaluation of these latter implications may both improve the design and conduct of LMIC HCS and/or inform ongoing debates in research ethics.^18^ Below, we provide preliminary analyses of issues that arguably warrant particularly careful attention.

### Generalisability

A key link between the scientific rationale and the social value of a study is the generalisability of the findings (whether these consist of knowledge of disease pathogenesis or estimates of the efficacy of a novel intervention) to the population and context for which the eventual benefits of a research programme are intended.^19 20^ The ethical acceptability of LMIC HCS might thus be contingent on such studies generating scientific knowledge that is particularly relevant to LMICs (ie, more relevant than knowledge that could be generated in HIC study populations) and/or testing interventions that would (if found to be effective and made available to relevant populations) be particularly beneficial to communities in LMICs.^19 20^

Certain choices in study design (eg, regarding the selection of participants, choice of challenge strain, etc) might lead to results that are more or less generalisable to the populations most at risk of a particular disease. For example, LMIC HCS would arguably be more generalisable and thus have a stronger scientific and ethical rationale in cases where there are (more) relevant features (eg, in terms of naturally acquired immunity, co-infections, genetics, microbiome, nutrition, etc) shared by potential (LMIC) study participants and the (LMIC) population(s) most at risk of the (locally endemic) infection in question.^15^ There will sometimes be difficult ethical trade-offs between the scientific aim to produce generalisable results and the protection of participants. For example, the use of wild-type strains in HCS may increase risks to participants (compared with the use of attenuated strains); but, assuming risks to participants can be adequately controlled, it may sometimes be more ethically justifiable to use wild-type strains where the use of other strains would provide less informative results (eg, regarding vaccine effectiveness). The need to determine an appropriate balance in such cases where ethical conflicts arise was identified as an unresolved issue and an area that arguably requires particularly careful attention in the evaluation of such studies.

### Benefit sharing

The sharing of benefits with study participants and/or local populations is commonly cited as an ethical requirement for international research involving human participants, but controversy surrounds questions regarding the nature, content and weight of such a requirement.^19–21^ How such a requirement is understood has important implications for the ethical justification of HCS conducted in LMICs. For example, a strict requirement for tangible benefits, such as vaccines (approved as a result of a study), could rule out *early phase* research (including some HCS designs, for example those exploring host-pathogen interactions and/or seeking to identify correlates of immunity) in LMICs because such research is not intended to lead to the immediate development or approval of such interventions.^19 20 22^ Nevertheless, such research can lead to important benefits in terms of scientific knowledge that is necessary for the development of such interventions in future. In particular, it can lead to knowledge that is of greater relevance to the population in question.^19 20^ Indeed, many of the LMIC HCS published in recent decades were focused on understanding locally relevant aspects of host-pathogen interaction without necessarily involving the testing of interventions such as vaccines.^23–30^ Thus, in terms of benefits to be shared, it may be more relevant to consider the importance of generating locally relevant knowledge and the overall long-term expected benefits from a programme of research as a whole (rather than a single study in isolation)^31^ especially in cases where there are future plans to test potentially beneficial interventions (so long as there is reasonable confidence that the programme can/will continue).

## Risks and other burdens

Research participation can involve a range of burdens; and some HCS designs entail relatively high levels of burdens for participants (eg, studies involving higher risk pathogens and/or long periods of inpatient isolation and/or close monitoring of participants), especially in comparison with other studies involving healthy volunteers.^32^ Here, we use ‘burdens’ to capture all potential compromises of individuals’ interests that occur due to research participation, including exposure to risk, privacy infringements, restrictions of freedom of movement, loss of time, other reductions in well-being, etc.^33^ The following sections summarise the findings of our project regarding (i) upper limits to risks to participants in HCS in general, (ii) risks to participants in (LMIC) HCS in particular, (iii) other burdens of participation, (iv) the right to withdraw and (v) risks to third parties.

### Upper limit to risk to participants

Recent HCS designs are generally considered to be safe. They have been approved by research ethics committees; there have been no reported deaths among participants; and significant lasting harms to participants are extremely rare (although, as discussed later, there may be some degree of publication bias, eg, regarding postinfectious complications).^34 35^ Many HCS designs nonetheless involve a relatively high probability of (short-duration) severe symptoms.^27 35 36^ However, HCS with higher risk diseases (perhaps including COVID19) may nevertheless be justified so long as the risks are minimised and outweighed by important benefits (e.g. the acceleration of vaccine development). Minimisation of risks to participants during HCS might involve, *inter alia* (i) selection of study participants at lower risk of severe disease, (ii) use of challenge strains that produce less severe disease, (iii) early diagnosis and/or treatment, (iv) keeping participants as inpatients to enable particularly close monitoring for at least part of the study, (v) close monitoring of (any) outpatient participants and/or (vi) careful follow-up for long-term outcomes. Many of these means of reducing risks frequently entail increasing other kinds of burdens (eg, such as those related to the close monitoring and/or frequent testing of participants) and/or a reduction in generalisability (eg, because selecting participants at low risk of severe disease might not produce results that are generalisable to those at higher risk). There may thus often be complex ethical trade-offs involved in study design.

With risk minimisation strategies in place, some HCS designs could plausibly be described as minimal risk (eg, those involving very low risk challenge strains and/or very early treatment), under a range of definitions of this term.^12^ Indeed, lower risk HCS (eg, many study designs in current use) might well be as safe or safer than many Phase I drug trials—which sometimes involve risks that are at the higher end of what might be considered acceptable for research involving healthy volunteers who are not expected to benefit directly from study participation.^37 38^ On the other hand, higher risk challenge designs might involve risks that *exceed* what might commonly be thought to be acceptable limits in research with healthy volunteers who are not expected to benefit directly from study participation. For example, cholera HCS in North American volunteers led to some participants experiencing >13 L of diarrhoea (whereas Thai participants in similar studies had up to approximately 3.6 L—perhaps due in part to immunity from prior infection).^30^ Higher risk pathogens might likewise involve small risks of death, although these can also be minimised either through early curative treatment (e.g. for malaria) or optimal supportive care (e.g. for dengue or COVID-19).

Aside from well-described risks, HCS participation sometimes involves significant uncertainty, particularly at the beginning of a research programme involving a novel HCS design (and/or new challenge strain) via which few (or no) people have yet been challenged. While HCS conducted under situations of uncertainty (at the outset of a research programme) might eventuate in unexpected harm, they can also yield scientifically important (but unexpected) results; and HCS research has revealed new and/or unexpected features of certain diseases.^39–41^ For example, a vivax malaria HCS revealed that some individuals are more likely to relapse (ie, develop recurrent infection) after treatment, resulting in (i) unique findings that inform vivax malaria treatment and public health programmes as well as (ii) some degree of (unexpected) harm to those participants in terms of a relapse of malaria.^39^ In situations of uncertainty, potential harms of HCS need to be weighed against potential benefits--but uncertainty *per se* presumably shouldn’t ethically preclude HCS.

There is no universally agreed-upon upper limit to acceptable risk in ‘non-therapeutic’ research with healthy volunteers that do not have potential to benefit directly from research participation. Proposals include (i) no limit,^42^ (ii) the risks of high-risk, socially beneficial occupations like fire-fighting^43^ or (iii) the risks of certain altruistic acts in a clinical context such as kidney donation.^44^ Some argue that it is more justifiable to pursue higher risk research where there is a high likelihood that a given study will produce significant benefits—but most commentators in the literature (and experts interviewed for this study) agree that easily imaginable HCS with very high risks^4^ would not be justifiable.^12 44 45^

A pragmatic reason for excluding very high-risk HCS is that the public reaction to severe harm that might otherwise result could lead to a moratorium on HCS—including those that are potentially beneficial but involve low risk as well as those that have very high expected benefits that outweigh significant but acceptable risks.^12^ Furthermore, at a local level, community acceptance of a given study design is arguably a necessary condition for the ethical acceptability of conducting such research in that community.^46^ Thus, although an absolute upper limit to risk for HCS in LMICs might be difficult to theoretically determine and/or defend, the setting of limits in particular contexts should be informed by community engagement, which is important to maintaining public trust in research (see online supplementary appendix, box 1).

### Long-term risks and lasting harms

Most commentators have argued that, as a general rule, HCS should involve infectious diseases that are treatable and/or self-limiting—that is, resolving in the absence of treatment, without causing serious harm.^1 10 11 45^ Some have added the criterion (and/or interpreted ‘self-limiting’ to imply) that there be no ‘lasting consequences’^45^ or ‘irreversible pathology’.^1^

Requirements that there be no lasting harms are potentially important since some infections (that have been or could plausibly be studied via HCS) do involve a low probability of lasting harm. Many of these risks can be reduced through careful participant selection, diagnosis, treatment and follow-up. However, while some postinfectious syndromes have known risk factors, meaning that individuals known to be at higher risk can be excluded from HCS, such strategies often cannot prevent these outcomes entirely (table 2). The fact that lasting harms have not been documented among modern HCS participants for these pathogens may reflect careful selection practices and/or relatively low numbers of total participants (in whom, by chance, a rare event has not been observed) and/or publication bias (ie, events that may have in fact occurred might not have been published). One HCS researcher interviewed was aware of at least one case of presumed postinfectious arthritis occurring after HCS with an enteric pathogen. (The case remains confidential and unpublished, consistent with publication bias.)

More generally, interviewees agreed that such lasting harms were particularly concerning and, at a minimum, require (i) careful risk mitigation strategies, (ii) long-term follow-up of participants and (iii) systems to compensate any participants who experience harm. However, there was no clear consensus about what level of residual risk of such outcomes, if any, should be considered acceptable (see online supplementary appendix, box 2 and table 2).

### Risks to participants in endemic LMIC settings

Conducting HCS in LMICs where diseases of interest are endemic might influence the potential risks to participants in several ways. On the one hand, where local health and related infrastructure is fragile, HCS participants in outpatient studies may encounter higher risks due to delays to treatment.^36^ It may sometimes be possible to mitigate risks such as these via inpatient study designs and/or capacity building of treatment centres. On the other hand, HCS in LMICs could involve lower risks of severe disease if they involve recruitment and infection of individuals who have (partial) acquired immunity due to prior infection and/or innate forms of resistance to particular pathogens (eg, genetic conditions that reduce the severity of malaria, which are much more common in endemic LMICs).^28^

Furthermore, where participants in a challenge study are already at risk of being infected with a pathogen in daily life (eg, because they live in an endemic area or because there is an ongoing or emerging pandemic), one might think that, in some cases, this background risk reduces the marginal risk an individual would take on by participating in a challenge study.^5^ It may thus be more ethically acceptable, from the point of view of balancing the risks and benefits of a study, to enrol those who already face higher background risk. In our qualitative research sample, there was widespread agreement among interviewees that such considerations might often favour conducting HCS in endemic populations (see online supplementary appendix, box 3).

Research ethics literature regarding background risk more generally,^47 48^ however, provides reasons for being wary about the sentiment that risk imposition on participants might be more acceptable where background levels of risk are higher when/if (i) higher levels of background risk themselves reflect injustices (eg, in LMICs) and/or (ii) research participation would significantly increase risks to participants who already face high background risks (while it should be kept in mind that *the absolute magnitude of risk increase is a key consideration*, independent of background risk magnitude).^18^

### Other burdens for participants

Since HCS frequently involve multiple study visits, blood draws and (inpatient or outpatient) monitoring by study staff, and since inpatient HCS in particular involve significant time away from normal activities, many HCS designs can be highly burdensome for participants. In some cases, such burdens might include adverse effects on mental health (eg, due to longer periods of isolation). Recent social science work with participants has suggested that HCS participation, particularly in inpatient studies, can lead to a wide range of burdens.^49^ Certain study interventions will be more burdensome for some individuals/populations than for others. Due to cultural beliefs regarding the value/importance of blood, for example, blood draws (especially of larger volumes) may be especially worrisome, and thus burdensome, to some research participants in sub-Saharan Africa.^49 50^ Many stakeholders we interviewed indicated that the burdens of participation (beyond risk imposition) raised ethically important issues for HCS design in need of further analysis (see online supplementary appendix, box 4).

### Right to withdraw

Certain participant behaviours might lead to greater risks than those anticipated in the study protocol—for example, participants (i) choosing to withdraw from the study and/or (ii) absconding (ie, leaving the study site and/or becoming uncontactable after being infected).^6^ Since they are exposing healthy volunteers (and sometimes third parties) to potentially severe harms (eg, if a serious infection went untreated), researchers arguably have weighty ethical obligations to ensure that such risks are minimised (whether or not participants themselves also have an ethical obligation to abide by monitoring, treatment and/or social distancing requirements during and/or after the study).

The right of participants to withdraw from a study—at any time, for any reason, and without having to give a reason—is widely endorsed in research ethics theory and practice, although the practical implications of this right have been a matter of debate in particular contexts.^51–54^ If challenge study participants were to exercise this right after being infected with certain pathogens, this might increase risks to the participants themselves and/or risks to third parties (which might be especially significant in LMIC contexts, as discussed below). Investigators have tried to address such dangers in study protocols, and in some cases public health authorities may be involved in HCS risk assessments and/or minimisation strategies. How exactly this kind of problem should be managed, however, remains an unresolved issue in need of further analysis (see online supplementary appendix, box 5).^55^

### Risks to third parties

Many types of research with infectious diseases can pose risks of infection to those not directly participating in the research (ie, third parties).^56–59^ In the case of HCS, the principal third-party risks are related to transmission of the challenge strain(s) from infected participants to others—and potentially onwards to many more people.^10–12 14^ Risks to third parties include the possibility of transmission (and/or harm) to unborn children, providing a reason to exclude pregnant women from HCS.^14^

Some have argued that researchers have extensive ethical duties to third parties where such risks of being infected by research participants exist. For example, some have argued that informed consent should be sought from at-risk individual third parties and/or local communities prior to study commencement.^56^ One way to obviate the need for such additional consent procedures is to reduce third-party risks to near zero by (i) rigorous biosafety procedures at HCS research centres and (ii) strict isolation of participants (eg, by keeping them in an ‘inpatient’ setting for the contagious period), although this entails significant burdens for participants. Alternatively, where such third-party risks are not minimised they could in some cases be monitored and/or quantified by (enhanced) public health surveillance in the local area including genotyping of strains detected (by surveillance) to assess potential spread of the challenge strain in the wider community.^60^ This, however, might significantly increase study costs and require capacity building of local public health laboratories in LMICs.

In any case, the potential risks may vary in different contexts and with different modes of disease transmission. For vectorborne diseases (VBD) such as malaria, if there are no local vectors then there are minimal risks of third-party transmission (apart from blood donation by participants while infected). Many ‘endemic’ LMICs contain regions or cities with no vectors; and, with respect to third-party risk, these are arguably ideal locations for VBD HCS research.^25 27 36 61^ The risks of transmission of diarrhoeal pathogens via sewerage systems could be higher in communities with poor access to sanitation, suggesting a strong rationale for inpatient studies, which have been the norm in enteric LMIC HCS in Thailand for example.^62 63^

The potential for higher third-party risks in endemic, epidemic, and/or pandemic contexts can lead to controversial questions. How important, for example, is a small third-party risk and/or single episode of transmission (eg, from a study participant to a third party) in the context of high local disease transmission (and/or high average local levels of immunity)? Some individuals (and communities) may consider this additional risk negligible (and some interviewed experts from both HICs and LMICs held this view), while others may see each additional episode of transmission as highly (ethically) significant (see online supplementary appendix, box 6). Given this potential controversy, and the potential for third-party risks to undermine public trust in research, such risks would constitute an additional reason for community engagement (to assess community views on the acceptability of such risks and/or to seek community consent for the research to proceed) and for carefully designed research procedures that reduce transmission risks).

## Participant selection, consent and payment

### Low-income and middle-income country populations

Calls for more LMIC HCS arguably go against the idea that, insofar as they are considered ‘vulnerable’ due to poverty and/or other factors, LMIC populations should be excluded from research, particularly early phase studies. One concern regarding exploitation of vulnerable populations is that relatively privileged populations are often the primary beneficiaries of research conducted in more underprivileged populations.^22^ However, conducting HCS in LMICs may be less exploitative in this regard if (i) the findings from such studies are more relevant to the (LMIC) populations in which the pathogen being studied is endemic and (ii) any scientific knowledge and/or new interventions produced by HCS are ultimately made available to those populations.^19 20^

An increasingly recognised problem is that excluding vulnerable populations from research as a way of protecting them from further burdens can ultimately lead to these same populations being excluded from the benefits of research.^64^ It may thus sometimes be ethically important to include vulnerable populations (with appropriate measures to minimise burdens), especially where the results of research in other populations are not likely to be generalisable to the vulnerable populations in question. This is one consideration that sometimes favours conducting (more) HCS in LMICs.

### Consent and education level

It is sometimes thought that it would be more ethical to recruit those with higher levels of education as research participants because this may improve (participant understanding and thus) informed consent. This is one reason why some LMIC HCS have aimed to recruit university students in particular.^26 64^ Despite these apparent advantages, there are also several ethical disadvantages of such a recruitment strategy: (i) excluding less well-educated individuals might be unjustified if they are able to understand a study well enough to provide adequate informed consent, (ii) university students (or those who have received university education) may not be representative of the eventual target population for an intervention (eg, because they are more likely to be affluent and/or to live in cities rather than highly endemic parts of LMICs, and/or because in some countries women are much less likely than men to receive university education), (iii) excluding less well-educated individuals from HCS research may thus be unfair (eg, where less well-educated individuals are at higher risk of the disease in question), (iv) students may feel pressure to participate (eg, from academics within the faculty with an interest in the study) making consent less voluntary^65^ and (v) educated individuals (eg, healthcare workers) may sometimes actually be less compliant with study protocols than other potential participants (and thus undermine the scientific validity of the study and/or increase risks).^24^

### Children

One of the most controversial questions regarding HCS concerns when, if ever, it might be ethically acceptable to recruit children as participants in studies that could cause significant symptoms/disease among (child) participants. On the one hand, many pathogens of interest predominantly harm young children in endemic settings who would thus stand to benefit most from new vaccines (and thus the research in question). On the other hand, (i) HCS in carefully selected adult volunteers might in at least some cases be sufficiently generalisable to children at risk (and thus obviate the need to recruit children), (ii) children lack the capacity to provide informed consent to participation, (iii) challenge infection might sometimes be higher risk in children and (iv) public perceptions of the enrolment of children in HCS could undermine trust in HCS and/or research more generally.

For these and other reasons, the only recent HCS performed with children have used (live-attenuated) vaccine strains of micro-organisms as the challenge agent.^65^ ^7^ Disease-causing HCS (with more pathogenic organisms) have not, to our knowledge, been undertaken in children since the 1950s–60s when the (now infamous) Willowbrook hepatitis studies were conducted in the USA.^9 47 66^ Less well known cases from that time period also included trachoma challenge among blind children in Jerusalem. These children were selected as research subjects because they were not at risk of the main complication of trachoma (ie, loss of vision). It is reported that they ‘willingly cooperated’ and experienced few burdensome symptoms).^67^

There was widespread consensus among those we interviewed that, even if disease-causing HCS in children could be conducted safely, there should be a presumption against enrolling children in such studies (with HCS in adults and/or field trials in children as plausible alternatives to be tried first). Arguably, several requirements would (all) have to be met before (disease-causing) HCS involving children were initiated, including (i) a strong and very carefully described scientific rationale, (ii) international consultation (eg, with the HCS community and international organisations) and (iii) clear demonstration of local community acceptance. A particularly strong theme that emerged in our stakeholder interviews was the importance of public and/or community acceptance and the risk of undermining trust in (HCS) research (see online supplementary appendix, box 7).

### Payment of participants

Since HCS participants endure costs and burdens while contributing to projects that primarily aim to benefit others (eg, future people at risk of the disease), some level of payment might be considered ethically appropriate. Payment of research participants remains controversial, however, especially in studies involving (economically) vulnerable populations and/or high levels of payment.^68–72^ Importantly, payment may be intended as (i) reimbursement for costs incurred, (ii) compensation for harms (if they occur), (iii) compensation for other burdens, (iv) an incentive for participation, or some combination of all of these goals.^68^

Attitudes towards the payment of research participants vary across different cultures/countries, as do payment practices. Small reimbursements (eg, for travel costs) are relatively uncontroversial, and there is also a recognition that studies involving large time commitments (eg, many HCS) may warrant greater payment.^32 49^ The need for compensation for research-related harms (if they occur) is also widely endorsed, although current practices regarding such compensation vary considerably in different countries.^73^

Payment intended to compensate for other burdens and/or incentivise research participation, however, is more controversial.^68^ Such payments, and even high levels of payment, are viewed as appropriate in some countries (eg, the UK and the USA^71 72^), but they are less accepted in other countries and are sometimes (eg, in some Latin American countries) even proscribed by local regulations and/or norms. Recent HCS in LMICs have involved varying levels of payment, often indexed to local wages—for example, (i) in Kenya, up to approximately US$500 for an inpatient HCS,^36 74^ (ii) in Thailand, amounts approximately US$300 or more for an inpatient HCS and (iii) in Colombia, no payment apart from reimbursement for costs (associated with travel, etc).

Stakeholders we interviewed generally agreed that payment for participation in (LMIC) HCS was ethically acceptable, and that it should be proportional to the burdens of HCS participation (eg, inpatient studies would usually be more burdensome and thus may warrant higher levels of payment). However, determination of the appropriate method(s) for titration of payment according to the burdens of participation was an area that was considered contentious (see online supplementary appendix, box 8).

### Undue inducement

Undue financial inducement might (or might be perceived to) (i) undermine informed consent by impairing decision-making (leading, eg, to participants accepting more risk than they would usually accept and/or forsaking responsibilities to children and family members)^49 75 76^; (ii) lead participants to conceal important details of their medical or psychiatric history which might increase risks to them and/or undermine the scientific validity of the study^68 77^ and/or (iii) lead participants to ‘overvolunteer’ (eg, participate in multiple studies simultaneously, which could have similar deleterious effects). In contrast, some have argued that—because each of these ethical concerns can be appropriately remedied without removing financial payment—payment per se is not ethically problematic. So long as a given study or programme of studies is otherwise considered ethically acceptable (eg, the benefits of the research outweigh risks, etc), according to this latter view, even high levels of payment should be considered acceptable.^71 78^

Many of our interviewees were concerned about the potential for undue inducement to participate in HCS (in both HICs and LMICs) and acknowledged that poverty and cultural norms could alter local perceptions of payment for research participation. However, some also argued that this should not necessarily preclude the payment of participants in LMIC HCS. Indeed, as noted above, most stakeholders we interviewed agreed that the level of payment should be proportional to the burdens of participation. Several acknowledged the potential for undue inducement among economically vulnerable participants in HICs, emphasising that this potential issue was not unique to LMICs, but one that requires further work to determine fair levels of payment in different settings (see online supplementary appendix, box 9).

### Other ethical issues related to payment

In addition to undue inducement, there may sometimes be other ethically salient effects of payment. First, payment may lead to the disproportionate recruitment of impoverished individuals and groups, which would in some cases arguably be unjust (eg, if the disease under study did not primarily occur in similar populations).^69 79^ Second, high payment in some studies may undermine sustainable research practices and fair availability of research opportunities for other (types of) studies in the same community, an effect to which institutions in resource-limited settings may be particularly susceptible.^76^ Third, some worry that payment may change the way that researchers treat research participants and/or that participants will view themselves as being akin to employees as opposed to volunteers^76^ (see online supplementary appendix, box 10). Some fear creating an ‘underclass’ of research participants (drawn from underprivileged groups in society) in both HICs and LMICs^79^ and/or (as mentioned above) ‘overvolunteering’ (ie, individuals participating in research too frequently in order to receive payment).^80 81^ Further social science research (regarding participants) as well as transparent longitudinal data (eg, regarding the level of payment in different studies, the sustainability of research with variable levels of payment over time and overvolunteering^80^) would help to clarify the practical importance of such concerns (see online supplementary appendix, box 10).

## Governance

HCS are sometimes perceived to be an unusual and/or particularly sensitive type of research, and thus some commentators have recommended the development of specific ethical frameworks/guidelines/principles^11 59 82^ and/or special ethics review procedures, although others argue that HCS should be handled by standard ethics review processes and research ethics principles (see online supplementary appendix, box 11).

### Ethical frameworks for human challenge studies

With regard to specific ethical principles/guidelines/frameworks for HCS,^10 66 83 84^ stakeholders we interviewed held a wide range of views. Some researchers felt that scientific considerations related to particular pathogens (eg, specific risks associated with a given infection) were more important than specific ethical principles in order to assure the safe conduct of HCS (see online supplementary appendix, box 12). Among those who favoured development of specific ethical guidelines/frameworks for HCS, certain ethical issues were identified as potential candidates for inclusion, including (i) limits to risk to participants, (ii) third-party risk management and (iii) harm-benefit assessments of HCS as compared with alternative study designs (see online supplementary appendix, box 12).

### Potential models for special ethical review

Regarding potential special review procedures for HCS, different models have been proposed, including: (i) the appointment of a special committee (eg, a national committee) or subcommittee for review of all HCS (eg, with additional infectious disease expertise),^11 82^ or (ii) special review for *new* challenge models in particular (perhaps followed by more usual review for future use of that model, once it is shown to be safe and scientifically valuable),^11 14^ or (iii) usual review with particularly strict requirements for a prior systematic review, publicly available rationale and well-defined compensation for harm (all of which might be required for other kinds of studies, but could perhaps be more strictly required in the case of HCS).^11^

Ultimately, policymakers in any given jurisdiction will need to adopt a policy regarding HCS review that is apt for the local context. Ethically, the important outcomes (regardless of the policy chosen) might include that burdens, including risks (to both participants and third parties), are appropriately minimised; that public trust in research is maintained and that scientifically valuable, acceptably low-risk studies are not unduly impeded by excessively costly or slow review procedures.^57^

As noted in other sections of this article, public controversies regarding (HCS) research have the potential to undermine support/acceptance of (other) research and/or public health endeavours—domestically and/or internationally. Thus, in some cases, international consultation and/or appeal to international agencies (eg, WHO) may be appropriate. Whether or not a particular jurisdiction decides on specialised or standard review, international agreement on an ethical and regulatory framework for HCS may help to improve review and ensure that relevant issues are consistently addressed.^66^ ^8^ Stakeholders we interviewed held a range of different views, with some in favour of standard review procedures (see online supplementary appendix, box 11), whereas others favoured some form of specialised review (see online supplementary appendix, box 13). Finally, some raised potential problems with specialised review (see online supplementary appendix, box 14).

## Conclusions

There are ethical and scientific reasons in favour of conducting HCS in endemic and/or LMIC settings in order to address the persistently high global burden of infectious diseases, especially in disadvantaged populations. Careful attention to the ethically salient aspects of HCS design, relevant governance mechanisms and the acceptability of HCS among participants and communities will help to ensure the sustainability of this important area of research which will hopefully produce significant benefits in such populations. Insofar as this article focuses on HCS in (endemic) settings where diseases are present and/or widespread, much of the analysis provided is relevant to HCS (in HICs or LMICs) involving pandemic diseases including COVID19. Given the complexities of such studies, and the controversial and/or unresolved issues highlighted in this article, further work is needed by biological scientists, social scientists and bioethicists to support ongoing improvements in the ethical design, conduct and review of HCS.^9^

## Footnotes

Nevertheless, there might be some cases in which HIC HCS are adequately generalisable to LMIC populations at risk. Recent typhoid vaccine research, for example, has shown that challenge study results in non-immune adult UK HCS participants were highly generalisable to field trial results in children in endemic LMICs; see Shakaya *et al*. *New England Journal of Medicine* 2019;381(23):2209-18

Philosophical approaches to this particular issue are discussed in more detail in Hope and McMillan (2004) and in the full report for this project (Jamrozik and Selgelid, Springer Briefs in Ethics, 2020).

This point is also especially relevant to HCS with epidemic/pandemic diseases such as COVID19 (in HICs or LMICs).

Consider HCS involving HIV.

With the exception of pathogens such as dengue, for which the sequence of infections with different strains influences the probability of severe disease (see Selgelid and Jamrozik 2018).

See, for example, https://www.theguardian.com/society/2010/oct/19/malaria-trial-nurse-found

In such cases, exposure to the live-attenuated vaccine strain as a challenge agent would entail an extremely low risk of harm and perhaps even a net benefit to participants.

The authors discussed parts of the current work with attendees at a recent WHO guidance development meeting regarding HCS (see ‘Acknowledgements’ section).

This point is also especially relevant to HCS with epidemic/pandemic diseases such as COVID19 (in HICs or LMICs)
