# Supplementary material for: Human infection challenge studies in endemic settings and/or low-income and middle-income countries: key points of ethical consensus and controversy
Source: J Med Ethics. 2020 May 7;46(9):601–9. doi: 10.1136/medethics-2019-106001 (PMC7476299; doi:10.1136/medethics-2019-106001)
Supplement: Supplementary data [file medethics-2019-106001supp001.pdf]

Appendix

(To paper: 'Human infection challenge studies in endemic settings and/or low- and middle-income countries: key points of ethical consensus and controversy')

1. Methods

This project was informed by a review of the relevant literature as well as qualitative interviews with science and ethics experts. We sought to identify (i) areas of consensus among the experts we interviewed and/or in the research ethics literature regarding issues that are highly salient to challenge studies, (ii) contentious and/or unresolved issues warranting further analysis and/or particularly careful attention during the design and conduct of such studies, whether identified in our qualitative research or in relevant current debates in the research ethics literature, and (iii) aspects of current research practice that provide useful case studies for such debates.

1.1 Literature review

Our review of academic literature and regulatory documents was particularly focused on identifying (i) primary scientific papers detailing LMIC HCS from 1990-2018 (n=13), (ii) relevant historical examples of (other) HCS, (iii) regulatory documents or policy consultations specific to HCS (whether HIC or LMIC), and (iv) bioethical analyses of HCS and/or ethical issues relevant to HCS in LMICs.

Relevant articles published between 1700[1] and 31st December 2018 were identified through searches in the authors' personal files, in Google Scholar, and

[1] This date was chosen because smallpox inoculation (a forerunner of vaccination and, arguably, human challenge studies) was subject to increasingly systematic investigations in the 18th Century.

in PubMed. Articles arising from these searches and citations within those articles were reviewed. For LMIC HCS, we included primary publications that gave details of HCS methods and results; conference abstracts were excluded due to lack of detail. Searches were conducted in English and articles published in English were the primary resources. Where articles in other languages had translations of their abstract or article available in English these were also reviewed. The search strategy included the terms: bioethics, dengue, ethic*, cholera, challenge model, challenge study, controlled human infection model (CHIM), controlled human malaria infection (CHMI), histor*, human challenge, human infection study, malaria, regulat*, schistosomiasis, shigella, typhoid, Zika.

1.2 Qualitative research

1.2.1 Interviews and coding

Our research team conducted qualitative interviews with 45 participants. We initially recruited informants based on involvement in the conduct of recent HCS in LMICs, expertise related to HCS, expertise in research ethics, and/or involvement in the regulation and/or funding of HCS research. Many interviewees currently working in HICs had been involved in and/or had expertise related to LMIC HCS in particular. Further informants were recruited on the basis of "snowball" sampling, based on suggestions from the above informants at time of interview. As detailed in [Table 1], we recruited a diverse group of participants with a wide range of expertise. De-identified interview transcripts were coded thematically with a combination of pre-set and open coding. The research team, informed by the main aims of the study, agreed upon an initial code list. Coding then progressed openly and iteratively as emergent codes arose and coding categories were further refined as agreed by the research

team. Data were organised and cleaned for use in the final analysis and report. As part of the consent processes, interview participants consented to be quoted anonymously (by pseudonym) in this report and other relevant publications and/or to waive the right to anonymity and be quoted by name.

1.2.2 Synthesis and validity checking

The findings of the literature review and thematic analyses of qualitative data were synthesized in the Final Report. Draft copies of the Final Report were shared with (i) a subset of participants who provided feedback to the research team (enabling an assessment of internal validity) and (ii) participants at two international meetings of researchers and policymakers with relevant expertise (enabling an assessment of external validity and transferability)[2]. Comments were incorporated, in most cases with de-identified acknowledgement in light of participants' wishes.

2. Qualitative Interview Data (quotes arranged by topic)

[Box 1: Limits to risk and public acceptability of research]

"[T]he way I understand [limits to risk in the context of ethics review] … is that … we're not just trying to make sure that this specific study is done well and it's ethical – we're also, to some extent, trying to protect the institution of research." [Ethicist, North America]

"I also think that those limits ought to be dictated by public perception, to a certain degree … [I]t's not merely a question ... [of] how much can we ask an individual to put their lives at stake; it also really bears on how much is the public willing to view this as a kind of legitimate and sanctioned activity, if we

[2] We are particularly grateful to participants at the June 2019 (i) Workshop: An ethical framework for human challenge studies (organized by A/Prof Seema Shah) and (ii) Guidance Development Meeting regarding the ethics of human challenge studies (convened by the WHO Global Health Ethics Unit)

put people at this level of risk." [Jonathan Kimmelman, ethicist, Canada]

"[W]hat we've learned in my [African] setting, and this is also looking back at some of the studies done here (which we thought were very safe) and how they became problematic … [W]e've learnt that we don't take anything for granted … in the community. We just have to be very careful about it, because it's got the potential to be misunderstood … in all different ways. It doesn't matter whether it is the most safe procedure you thought you were introducing; as long as it is unfamiliar in the community, it is likely to flare up all kinds of rumors." [Scientist, Africa]

[Box 2: Lasting and/or irreversible harms]

"I'm very uncomfortable with the idea that you might leave somebody with irreversible harm when they haven't been given any possible benefit." [Scientist, UK/Europe]

"[I]f there's irreversible harm, I think most people would say that that's unreasonable risk … [T]here may be risk of severe injury in some human challenge studies; but, if the risk is very low – one in a million, one in a hundred-thousand – perhaps then that might be considered a reasonable risk. But that's where it does get into judgement." [Regulatory representative]

"[S]ome of these infections or some of these infection models [involve] risks that you don't know about and you can develop chronic consequences after these infections. And so I think … we need to fully inform [potential participants] that it's not just … this acute infection, that it could lead to something chronically" [Scientist, North America]

"The borderline cases [ethically speaking] are [firstly, challenge studies] where the diseases are serious and people get really sick ... and the second kind is where there are these long term effects that aren't entirely predictable. To me those are kind of like two features of the borderline cases." [Ethicist, North America]

[Box 3: Background risks of infection and risk to participants]

"[I]t's less ethically difficult in recruiting volunteers [in endemic areas], considering that you're giving someone an infectious disease, to use volunteers drawn from a population that's at risk anyway, rather than a population that would never be at risk, in terms of justifying the balance of risk" [Scientist, Asia]

"[I]f you are already exposed, if you're at a greater risk, [the risk] you're being asked to accept as a result of your … participation in the study is lower and the benefit is going to be the same. So the benefit versus risk profile [is better].", [Ethicist, North America]

[Box 4: Burdens of participation]

"[HCS protocols often] keep people in residence for a long period of time. I think that's pretty unique. I don't think we do that for many other studies … just that phenomenon of saying to people – you know, you might need to be in residence for a month or even longer, six weeks, you'll need to stay here and you will not be able to leave, under any circumstances … I don't think we fully understand what the ethical implications of that are." [Scientist, Africa]

"[T]here's physical risk, which I think for [some HCS] is quite small, but there is also the emotional risk … but the bigger thing is the burdens. [In some HCS designs] you have to be in residence for fourteen days, minimum, [and] being in residence means that you have to make sure that other parts of your life … and kids, and jobs [are taken care of] … so that's quite a big commitment, and a sacrifice, I would say." [Scientist, Asia]

"How can we make [participation a good] experience good for them? What kind residence would that be required to be? … If we are curtailing their freedoms of movement, how does that then balance against the risk? … We are telling them not to go to endemic areas, even when they do get out of there – they're still within the study. In other words, we're interfering with their freedom, and how do we take account for that?" [Scientist, Africa]

[Box 5: Participants' right to withdraw]

"[T]hese are adults and … their participation in the study is voluntary. They could always withdraw their informed consent. They could walk even though that is a risk to the community at large. We can't hold them against their will. And that was a concern. And so we spent a lot of time emphasising to them … early on in the trial, how important it was that they complete the treatment and they complete the follow-up. And that we understand that it's a long time." [Carl Mason, scientist, USA]

"I think if there was a scenario where somebody left and they wouldn't take the treatment … we would have to let them go. We did say if somebody went missing … we would notify the local authorities to search for them because there would be a concern about their mental and physical health. And we would contact their next of kin … Of course, in the UK, the concern [in malaria HCS] is not about transmission, it's about wellbeing of the patient. But, you know, in Nairobi [given the local presence of vectors] there's a potential to have onward transmission as a result of not having treatment." [Scientist, UK/Europe]

"Another issue I see as more complicated in the context of challenge trials is the right to withdraw … There are other trials where you still can't quite just leave when you want to leave because it may not be safe for you, but challenge trials are trials where that issue becomes difficult. And I think it would be helpful to

know more, to have a better public health framework maybe similar to what we think about for quarantine, to understand what [are] the limits of measures to restrict someone's liberty, if they're in a challenge trial"
[Ethicist, North America]

"[W]e had someone in one of our typhoid studies who absconded because he was an actor and had an audition, which he hadn't been expecting, for a lead role in a play … [W]hilst he was developing typhoid he went to do his audition, and we lost touch with him and we were very worried about him and in fact he got the role … and he actually had positive blood cultures for typhoid at the time."
[Scientist, UK/Europe]

"[W]e had one volunteer leave from one of our studies … [H]e'd been challenged as part of that study and he decided he'd go and see his uncle [in another country]. And fortunately we caught him and we made sure he took [treatment for his infection], and so on" [Scientist, North America]

"There was talk of once in a while somebody sneaking out and going to the shops but not much of like, somebody being away in terms of going home and putting others at risk … Somebody will sneak out of the gate of the university and go to the shops nearby and come back." [Scientist, Africa]

[Box 6: Third party risk and background risk]

"I think it really depends on the background transmission rate … [I]f you're working in a hyper endemic setting, I just don't think there's any quantifiable increased risk to the population … [P]eople are being infected every week, all the time, so … I don't think that's a risk, a real increase[d] risk for the population."
[Scientist, UK/Europe]

"[If] there is not much greater risk [to third parties, compared to background risk] and you are not using a strain that is resistant to any of the drugs that are available, then people [once they understand this] will be much more comfortable I think ... most of the risk that we see are much more academic than real [or] practical" [Scientist, Africa]

"[C]ontainment is possible. It's expensive. Not so expensive in developing countries as it is in developed countries, but it's possible and if you can minimise risk [to third parties] you should do so, and remember that it's a drop in the ocean, but it's a drop in the ocean that can result in death." [Scientist, UK/Europe]

"I think of the response if an individual is inadvertently infected. A third party individual is going to [feel] different[ly about it] if they later learn that it's because they came into contact with someone who was in a scientific experiment, than if it's [just] because of a mosquito … [R]isk is, or has, these … moral layers … that we all … bracket when we talk about risk … in a quantitative way." [Jonathan Kimmelman, ethicist, Canada]

[Box 7: HCS in children]

"[A]s a researcher, I would say you could do [a malaria HCS in children with early treatment] safely, but getting it past an ethics committee would be a massive challenge. Certainly, you know, giving kids malaria … the optics of it are not good. So, it would have to be preceded by a massive public and stakeholder engagement campaign." [Scientist, Asia]

"I think we might be able to justify [a *Shigella* HCS vaccine trial in children] but it would … definitely be on a case-by-case basis and there would have to be tremendous consensus both in the host country probably as well as globally. That's something you would have to take to [a] body like WHO and really try to build a consensus." [Carl Mason, scientist, USA]

"[Y]ou know the difficultly of doing vaccine research in children and you just have to have a few things, coincidently, go wrong and you can destroy a whole program of research or public health implementation." [Scientist, UK/Europe]

"I think … in general most people would say that it's just … unacceptable to do challenge studies in children. I think that's most people's starting point, and I think before we move away from that position, we'd have to be on really very solid ground … I hope nothing like that proceeds without all sorts of very extensive consultations and discussions." [Scientist, UK/Europe]

[Box 8: payment in LMICs]

"[T]he current situation is … for whatever reason, [that] somebody says it's not appropriate to pay people in developing countries ... and sometimes that didn't come from developing countries themselves, it comes from somewhere else" [Scientist, UK/Europe]

"[I]n endemic countries I think … when you're asking people to give up ten days of their life, to stay in an inpatient unit … I think they should be compensated. That's … a lot of their time and freedom. Certainly it's time away from how they could be making other money – and it's difficult, frankly, to find people altruistic enough [to] say 'Sure, I'll stay in your inpatient unit for ten days, for the betterment of science.'" Prof. Anna Durbin, scientist, USA

"[F]or a long time, in a [low-income] setting [the standard view has been that] people should not be compensated, so that they can make a voluntary decision not driven by gains that might accrue from participating in the study … [A]s much as people get worried about [payment in LMICs], it is the same as what you are seeing with people who are doing the phase one studies in Europe … [T]he students end up doing that [i.e., serving as participants], because they want some extra money [and] because they want to be a part of something." [Scientist, Africa]

"[H]ow do we appropriately compensate for all these inconveniences? … Are we compensating for the fact that the inmate actually gets sick and feels all the discomfort that comes with the sickness? How do you even know what to

compensate for that? You can compensate for time stayed, you can compensate for expenses, you can fund expenses, but how do you compensate for someone being sick?" [Scientist, Africa]

"I don't want to underpay people because it's not fair to underpay people. If I take two weeks out [to participate in research], committing this amount of time and sacrificing my social life, [and] probably [drinking] no alcohol for two weeks, [that would constitute a significant burden]. Come on, you have to pay people!" [Scientist, Asia]

"Often the procedures for challenge studies are really quite onerous compared to other studies so if you just add all that up together, just logically, the amount that they should be paid is more than for other studies. How much that should be, should probably be linked to local purchasing parity. That makes sense to me." [Scientist, UK/Europe]

[Box 9: Undue inducement and vulnerable populations]

"[O]ur ethics committees are very worried about compensation and inducement … But if you look at challenge studies in the West, you look at challenge studies in Africa, whoever is [participating in] the study always says, 'My main motivation is the money.' But, has anybody set a price on what is enough, and what is insufficient? … [I]f I paid someone $100 a day in Baltimore would they participate? If I paid them $1000 a day in Baltimore would they participate? Where do you set the price? … In India or in Africa or anywhere else, I think you should compensate people who are volunteering, and I think you should compensate them well. Inpatient studies should definitely be compensated more than outpatient studies but ... I don't think we've done enough work on what's right, and what is actually inducement." [Gagandeep Kang, scientist, India]

"[T]he one worry I have is that if you pay people a lot of money that you could have a higher increase in people not revealing certain information to the study team and lying about inclusion criteria, exclusion criteria, or what they did in the course of the trial, or what side effects they're experiencing. And all of those things could have a detrimental impact on safety. So I think we probably need more data on whether higher payments induce people to conceal information that would relate to their own protection." [Ethicist, North America]

[Box 10: Sustainability, over-volunteering, and relationships between researchers and participants]

"There are institutions that are involved, and look at what should be acceptable … [W]ithin our setting, what can we possibly keep up with, and what can we sustain? … [O]nce you start compensating people at a … certain level, they will expect that to continue. And when it comes to another study they will not [have the same levels of compensation], so I think it's one way [of] looking at what would be acceptable within our frameworks, I think, if you are driven by local institutions [and their views on sustainable payment levels]" [Scientist, Africa]

“[T]here are several … phase one trials that are happening in India where we have these professional trial participants. They make a livelihood out of trial participation … [T]here is a washout period of 45 days or something and … after every … 45 days, they just go and participate in one trial after another; and these people, if there is no trial, if they are not eligible to participate in a trial, they go hungry.” [Vijayaprasad Gopichandran, ethicist, India]

“[Overvolunteering] will undermine the science, but I think the primary thing is … thinking from a more society than science perspective … [w]hat it winds up doing is giving all of research a bad name. So the fact that your own research got ruined is bad enough, but you are ruining then research for a number of different areas.” [Gagandeep Kang, scientist, India]

[Box 11: Standard ethics review of HCS]

“[A]t the end of the day, the requirement[s] for a trial just like any other trial are the same … I think the main thing is building the capacity of the ethics committees to know what are the issues around challenge studies, what are the salient issues and what are the emerging issues.” [Scientist, Africa]

“I think the important thing is that they’re reviewed by a committee with sufficient capacity to perform the review, full stop. It doesn't need to be a national committee. But there may be some settings where they don’t really have that capacity. They would need to be able to really understand this, and it actually relates to … a general issue about this review, which is how the scientific aspects are reviewed. [Importantly,] somebody needs to be able to look at the scientific basis for the risks and the benefits ... [The committee] needs to have adequate capacity [to review] both the scientific and the ethical … considerations … I would say that's the same for all clinical research.” [Scientist, UK/Europe]

“[Y]ou can imagine [that], in science, we’ll always have new things coming up … The issue is how do we capacity build the ethics of your committees to address the new changes that are coming in … proactively, not wait[ing] for things to happen, for them to catch up with how they review … I think there’s a lot of experience in ethical review, there’s a lot of capacity building that has been going on.” [Scientist, Africa]

[Box 12: Ethical frameworks / guidelines for HCS]

“I think we do need more frameworks and guidelines for human challenge studies because I think they do raise [particularly salient] questions … and, therefore, require more careful thinking than we’ve done in the context of other types of trials.” [Ethicist, North America]

“I wouldn’t make it a special framework specific to challenge studies. There are things that arise in challenge studies that might also arise in other contexts that I think call for a different framework. [For example,] bystander risks come up in challenge trials but also come up in other types of research like HIV cure studies. And [bystander risks] are not something that IRBs or the US regulations cover. So you do need a different framework to think about bystander risks.” [Ethicist,

North America]

“[HCS are] a challenge for our [standard research ethics] benefit framework. Is it really the case that, if the question is socially important enough … an adult can consent to, in effect, an unlimited amount of [or at least] a very high degree of risk? I think that can’t be right. So, I think there has to be a line there somewhere … What that line is to me, is really the hard question of [HCS].” [Ethicist, North America]

“I think the principles are largely the same as with other types of studies but there are these additional questions that we’ve actually been debating like [for example] if there’s a perfectly good animal model or you’ve got a very high attack rate in the field and you only need to recruit twenty people in the field, why would you deliberately expose healthy people to the pathogen? So I think there are issues around understanding challenge studies [and] the scientific process which leads into the ethical questions is quite important.” [Scientist, UK/Europe]

“[Ethics committees reviewing HCS] generally [have] some expertise in the disease that you’re working with and [disease-specific expertise] is, I think, more important than having a general framework for all the different challenge models. So I would rather put my protocol in front of a malaria specialist than a generalist in … challenge models … There are different risks for *Shigella*, for *Salmonella*, [and] for malaria – and to generalise those into one framework I think you run the risk of trivialising some of those risks or [by trying to] make a level playing field for everyone you’ll [make], say, *Shigella* [on par with] malaria when they’re really not on a par.” [Scientist, North America]

“I think it would probably be useful to have special guidance for low resource settings. I think there are just enough issues around how much infrastructure is enough infrastructure, payment issues, community consultation issues, [etc.]. So I think the idea of guidance that’s directed at low resource settings would be useful.” [Ethicist, North America]

[Box 13: Advantages of specialised ethics review for HCS]

“IRBs work really well for fairly routine … garden variety research, but I think when you’re at the vanguard, you want some sort of specialised mechanism and … I think there are substantive reasons for that, that get to the quality of the expertise that you get – and specialised review, the ability to pick through and second guess the scientific rationale, is really key.” [Jonathan Kimmelman, ethicist, Canada]

“I think the most important thing is to be transparent. So whatever process you set up – and perhaps having a central high-level review mechanisms for all such studies is the way to go – … to make it clear that there is nothing being hidden from anybody is the important thing. To some extent, having a centrally mandated committee would also be helpful because it would provide some distancing and protection from the investigators and their institutions.” [Gagandeep Kang, scientist, India]

"[T]here may be some cases where having an extra layer of review that the researcher either voluntary agrees to or that the sponsor puts in a place can help make sure that everything is done as rigorously and carefully as possible and then … reassure the existing levels of review that we already have." [Ethicist, North America]

[Box 14: Potential problems with special review for HCS]

[S]pecial challenge ethics committees … can give you clearance to do stuff which nobody else can and [that] doesn't sit right with me … [I]t should be … the general ethics committee and … they should have training and they should understand the issues and … of course, also I think investigators [should] make sure that the issues are clearly articulated" [Scientist, Asia]

"I suppose you could [have a specialised IRB for HCS] but they're so rare in a given place … and to set up something separate just for one study a year for challenge [studies] is a bit over the top I feel. It's an over-response to a problem that doesn't exist." [Scientist, North America]

"I'm not sure that having a panel of experts will speed anything up! … [W]e had difficulty enough just explaining the host country [i.e., LMIC] processes to [those involved in] the US regulatory review process … I would think that kind of requirement should probably come from the host country. If the host country wants to have [an] additional advisory [body] or ask the WHO, or ask some other group … it's really up to them to decide what level of review they think is necessary." [Carl Mason, scientist, USA]
